# Unusual Presentation of Von Hippel-Lindau Syndrome With Gastric Variceal Bleeding

**DOI:** 10.7759/cureus.64685

**Published:** 2024-07-16

**Authors:** David W Graham, Blake Thompson, Parvez Mantry

**Affiliations:** 1 Internal Medicine, Methodist Dallas Medical Center, Dallas, USA; 2 Gastroenterology, Methodist Dallas Medical Center, Dallas, USA; 3 Hepatology, Methodist Dallas Medical Center, Dallas, USA

**Keywords:** sinistral portal hypertension, chronic pancreatitis, von hippel-lindau disease (vhl), splenic vein thrombosis, gastric varices

## Abstract

Gastric varices are most commonly a complication of portal hypertension or splenic vein thrombosis (SVT). The presence of gastric varices due to portal hypertension is significantly less than the prevalence of esophageal varices. SVT is a known complication of pancreatitis due to inflammation or compression of the splenic vein coursing along the posterior surface of the pancreas. Occlusion of the splenic vein leads to left-sided portal hypertension. Left-sided portal hypertension results in the development of collateral vessels that bypass the splenic vein by connecting with the short gastric veins. The associated increased pressure within the gastric vessels results in gastric varices. Gastric varices due to SVT may occur in the absence of or be disproportionate to esophageal varices. We report an interesting case of gastrointestinal bleeding from gastric varices related to cirrhosis secondary to metabolic dysfunction-associated steatohepatitis and SVT secondary to chronic pancreatitis due to pancreatic neuroendocrine tumor (NET) in a patient diagnosed with von Hippel-Lindau (VHL) syndrome.

## Introduction

Von Hippel-Lindau (VHL) syndrome is one of the most frequently occurring familial cancer syndromes characterized by the development of benign and malignant tumors including hemangioblastomas of cerebellum and spine, retinal hemangiomas, clear cell renal cell carcinoma (RCC), pheochromocytomas, and pancreas tumors [1,2]. We present a patient with multiple manifestations of VHL including metastatic neuroendocrine tumor (NET) of the pancreas complicated by chronic pancreatitis who presented with upper gastrointestinal (GI) bleeding due to bleeding gastric varices.

Gastric varices are the result of vascular shunting between the systemic and portal circulation in the mucosa or submucosa of the stomach. Gastric varices are most commonly seen as a complication of portal hypertension or SVT causing left-sided portal hypertension. The presence of gastric varices associated with portal hypertension is estimated at 17-25% [3]. Upper GI bleeding secondary to gastric varices due to SVT is a relatively uncommon complication of chronic pancreatitis. SVT is most commonly caused by chronic pancreatitis and pancreatic inflammatory diseases. The course of the splenic vein is along the posterior surface of the pancreas. Inflammation in the setting of chronic pancreatitis may lead to SVT with an incidence of 12.4% of patients with chronic pancreatitis [4]. Autoactivation of pancreatic enzymes in pancreatitis may lead to auto-digestion, necrosis, or vasculitis. The vasculitis triggers an inflammatory process that may damage vessels near the pancreas and promote clotting [5]. Pancreatic NETs may compress the splenic vein and are a rare cause of SVT [6]. Left-sided portal hypertension should be considered in patients presenting with gastric varices without esophageal varices, splenomegaly, and no evidence of liver disease. Gastric variceal bleeding may be managed with pharmacologic therapy, endoscopic cyanoacrylate glue injection (ECI), or endovascular interventions. Isolated gastric varices due to SVT can be definitively managed with splenectomy.

This case report was previously presented as a poster at the American College of Gastroenterology 2023 Annual Scientific Meeting on October 22, 2023.

## Case presentation

A 45-year-old Hispanic male with VHL presented to the Emergency Department after sustaining a fall when he awoke to use the restroom. He also has a history of multiple renal cell carcinomas (RCC) status after left partial nephrectomy and right radical nephrectomy, adrenal pheochromocytoma status after resection, cerebellar hemangioblastoma status after two resections, and unresectable pancreatic NET with metastases to liver and lumbar spine complicated by chronic calcific pancreatitis and chronic splenic vein thrombosis (SVT) dating back to 2012. CT imaging at that time noted unremarkable liver without focal lesions, extensive gastric varices without esophageal varices, and multiple soft tissue masses in the pancreas that may have been contributing to the SVT due to mass effect. He had been previously seen in the Hepatology Clinic in 2015 for elevated liver enzymes, diagnosed with metabolic dysfunction-associated steatohepatitis with stage 3 fibrosis on liver biopsy, and had been undergoing treatment of VHL with octreotide and belzutifan. He reported melanic stools over several days.

**Figure 1:**
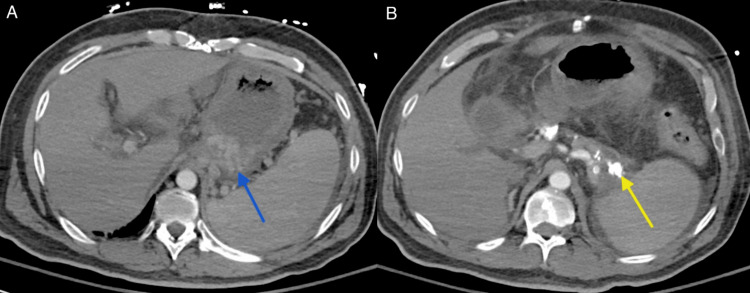
CT abdomen/pelvis with IV contrast A: Diffusely thick-walled and distended stomach with gastric varices (blue arrow) and splenomegaly. B: Extensive pancreatic calcification (yellow arrow).

He was tachycardic but normotensive. Labs were remarkable for white blood cell count of 12.2x10^3^/uL (3.8-10.6 x10^3/uL), hemoglobin of 4.7 g/dL (13.5-17.5 g/dL), platelet count of 106x10^3^/uL (130-400 x10^3^/uL ), AST of 64 U/L (8-42 U/L), ALT of 54 U/L (<50 U/L), ALP of 947 U/L (38-126 U/L), total bilirubin of 0.8 mg/dL (0.0-1.4 mg/dL), INR of 1.9 (0.9-1.2), and albumin of 3.1 g/dL (3.5-5.7 g/dL). Computerized tomography (CT) scan showed calcifications throughout an atrophied pancreas, SVT, portal vein thrombosis (PVT), a cirrhotic appearing liver with enhancing lesions throughout, splenomegaly, and gastric varices (Figure 1).

A hepatology consult was requested for suspected variceal bleeding in the setting of cirrhosis and SVT. Esophagogastroduodenoscopy (EGD) showed a large network of cardio-fundal varices (gastroesophageal varices type 2) with stigmata of recent bleeding. During the initial admission, he underwent endoscopic coil embolization of gastric varices, which involved intravascular delivery of coils with subsequent cessation of Doppler flow in the gastric variceal nest. The patient had no overt evidence of GI bleeding following the procedure, and hemoglobin remained stable throughout the remainder of his admission. He was discharged in good condition.

**Figure 2:**
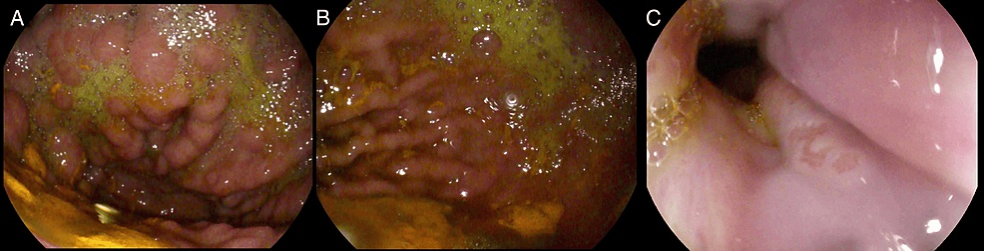
EGD after coil embolization A and B: Extensive type 2 gastric varices found in the gastric fundus. C: Examined esophagus was normal. EGD, esophagogastroduodenoscopy

However, embolization was ultimately unsuccessful, and the patient had several ensuing admissions at various hospitals in the area for GI bleeding. He was admitted again four months after his initial presentation for right foot cellulitis but was also noted to have melena. Follow-up EGD showed normal esophagus, extensive isolated gastric varices without signs of bleeding, and portal hypertensive gastropathy (Figure 2). The gastric varices were thought to be due to known chronic SVT. The patient was not a surgical candidate for splenectomy given his multiple comorbidities and not a candidate for anticoagulation due to the risk of hemorrhage. During this admission, the patient was evaluated for balloon-occluded retrograde transvenous obliteration (BRTO). To perform the BRTO, an occlusive balloon is advanced through the systemic venous outflow pathway, such as the left renal vein, into the portosystemic shunt with the intent of occluding the shunt and administering a sclerosing agent into the shunt and gastric varices [7]. In this case with a patient with SVT, the access would be through a splenorenal shunt. During assessment by interventional radiology, the evaluation showed that there was no splenorenal shunt present and thus no viable access to the gastric varices to inject a sclerosing agent. The patient was, therefore, not a candidate for BRTO. The patient underwent transjugular intrahepatic portosystemic shunt (TIPS) the following day and had no recurrence of bleeding during this hospitalization. His hemoglobin remained stable. The patient was not a liver transplant candidate due to an overall poor prognosis and multiple comorbidities.

He was hospitalized two months after discharge at another area hospital due to symptomatic anemia with hemoglobin of 4.4 g/dL due to an upper GI bleed. He underwent EGD at that time showing Grade I esophageal varices, gastric varices, and moderate to severe portal hypertensive gastropathy thought to be the source of bleeding. Ultrasound of TIPS at that time showed complete occlusion. He underwent TIPS revision with interventional radiology. A follow-up ultrasound one month later showed a recurrence of complete thrombosis of TIPS. He again had successful TIPS recanalization with balloon maceration of in-stent thrombosis with significant improvement in portosystemic gradient. Outpatient follow-up visits showed stable hemoglobin. The patient ultimately died 11 months after the initial presentation. The circumstances and cause of death are not available in his electronic medical records.

## Discussion

VHL is a hereditary, autosomal dominant cancer syndrome with a penetrance of over 90% by the age of 65. VHL is characterized by the development of multiple benign and malignant vascular tumors. Incidence is approximately one in 36,000 individuals [1,2]. The pathogenesis of VHL occurs due to a "two-hit" model resulting in a germline mutation of the VHL tumor suppressor gene. This causes an increased production of VEGF, PDGF, and TGF-α, resulting in vascular tumors affecting the retina, brain, and spine along with benign and malignant tumors or cysts of visceral organs [1]. VHL disease is associated with hemangioblastomas of the cerebellum and spine, retinal hemangiomas, clear cell RCC, pheochromocytomas, pancreatic islet cell tumors, endolymphatic sac tumors, and cystadenomas of the broad ligament or epididymis. VHL is broadly divided into Type I and Type II and can be further subdivided based on the presence of hemangioblastoma and RCC. Type I VHL is associated with large deletion mutations resulting in decreased or no functionality in the encoded protein. Type I VHL is associated with CNS and retinal hemangioblastoma and RCC with a low risk of pheochromocytoma. Type II VHL is often associated with missense mutations resulting in an encoded protein with diminished functionality and results in a higher risk of pheochromocytoma [2]. Diagnosis of VHL involves clinical suspicion and is confirmed with molecular testing. Clinical presentation of individuals with VHL is variable depending on the system affected. Treatment consists of regular surveillance, resection of tumors, or treatment with HIF-2α inhibitor, belzutifan [8,9].

Our patient had multiple organ systems affected, including NET of the pancreas resulting in chronic calcific pancreatitis with associated SVT complicated by bleeding gastric varices. Compression of the splenic vein due to the mass effect of the pancreatic NET may have also been a contributing etiology for SVT in this patient. Gastric varices arise most commonly from portal hypertension or SVT. Portal hypertension is commonly caused by cirrhosis. Increased vascular resistance leads to elevated outflow resistance in the portal sinusoids. Elevated portal pressure induces splanchnic vasodilation and increased portal inflow. Portal hypertension induces portal-systemic collaterals and leads to the development of varices [10]. SVT most commonly occurs in the setting of acute or chronic pancreatitis due to obstruction or inflammation and damage to the splenic vein as it runs along the posterior surface of the pancreas. The occlusion of the splenic vein results in left-sided, or sinistral, portal hypertension. The mechanism of gastric variceal formation due to left-sided portal hypertension involves collateral vessels connecting to the short gastric veins to bypass the occluded splenic vein. Isolated gastric varices in the absence of esophageal varices are strongly suggestive of SVT, as collateral vessels that connect to esophageal veins can drain into the left gastric vein [11]. Initial management of a variceal bleed involves resuscitation along with pharmacologic or endoscopic hemostasis. Pharmacological treatment involves treatment with vasoactive medications to reduce splanchnic blood flow. Endoscopic hemostasis may be achieved by ECI. Following endoscopic hemostasis, cross-sectional imaging with portal venous contrast phase should be obtained to determine the presence of portosystemic or gastrorenal shunts. When present, BRTO, plug-assisted-retrograde transvenous obliteration (PARTO), or transhepatic coronary venous embolization are effective therapeutic options [3]. In those with significant portal hypertension, TIPS is an option. In those with SVT, eventual splenectomy is the treatment of choice [11].

## Conclusions

The case highlights one of many presentations of VHL and the challenges posed in the management of these patients. This patient had several manifestations of VHL including RCC, pheochromocytoma, hemangioblastomas, and pancreatic NET complicated by chronic pancreatitis causing SVT and gastric varices with a likely component of portal hypertension secondary to cirrhosis contributing to the formation of gastric varices. While this patient did have observed isolated gastric varices in the setting of SVT visualized on CT imaging dating back to 2012 prior to any evidence of cirrhosis, he likely progressed to cirrhosis by the time of his presentation with upper GI bleed in 2023. It is likely portal hypertension due to cirrhosis contributed to the recurrent variceal bleeds in addition to left-sided portal hypertension due to SVT. Our patient was not a candidate for BRTO due to a lack of access to inject a sclerosing agent into the gastric varices without a splenorenal shunt present. He was also not a candidate for splenectomy or liver transplant due to his multiple comorbidities and overall poor prognosis and was ultimately treated with TIPS and recurrent recanalization. Patients without liver disease presenting with gastric varices without esophageal varices should be considered for evaluation of left-sided portal hypertension. Future research may focus on non-surgical or less invasive surgical management options and improved diagnostic methods.
